# Measurements of the parapapillary atrophy zones in en face optical coherence tomography images

**DOI:** 10.1371/journal.pone.0175347

**Published:** 2017-04-17

**Authors:** Atsuya Miki, Yasushi Ikuno, Robert N. Weinreb, Junko Yokoyama, Tomoko Asai, Shinichi Usui, Kohji Nishida

**Affiliations:** 1 Department of Ophthalmology, Osaka University Graduate School of Medicine, Osaka, Japan; 2 Hamilton Glaucoma Center, Department of Ophthalmology and Shiley Eye Insititute, University of California, San Diego, La Jolla, California, United States of America; 3 Department of Ophthalmology, National Hospital Organization Osaka National Hospital, Osaka, Japan; Purdue University, UNITED STATES

## Abstract

**Objective:**

To measure the parapapillary atrophy (PPA) area in en face images obtained with swept-source optical coherence tomography (SS-OCT), and to evaluate its relationship to glaucoma, myopia, and age in non-highly myopic subjects.

**Design:**

Retrospective, cross-sectional study.

**Participants:**

Fifty eyes of 30 subjects with open-angle glaucoma (G group) and forty-three eyes of 26 healthy control subjects (C group). Eyes with high myopia (spherical equivalent refractive error ≤ -8 diopters or axial length ≥ 26.5 mm) were excluded.

**Methods:**

Mean age ± standard deviation was 59.9 ± 12.4 years. The beta zone and the gamma zone PPA areas were measured in en face images reconstructed from three-dimensional SS-OCT images. Relationship between the PPA areas and patient characteristics such as glaucoma, axial length, and age was statistically evaluated using multivariate mixed-effects models.

**Main outcome measures:**

Areas of the beta zone and the gamma zone PPA measured on en face OCT images.

**Results:**

Average ± standard deviation area of the beta and the gamma zone was 0.64 ± 0.79 and 0.16 ± 0.30 mm^2^, respectively. In multivariate models, the gamma zone significantly correlated with axial length (P = 0.001) but not with glaucoma (P = 0.944). In contrast, the beta zone significantly correlated with age (P = 0.0249) and glaucoma (P = 0.014).

**Conclusions:**

En face images reconstructed from 3D SS-OCT data facilitated measurements of the beta and the gamma PPA zones even in eyes with optic disc distortion. The OCT-defined beta zone is associated with glaucoma and age, whereas the gamma zone correlated with myopia but not with glaucoma. This study confirmed the clinical usefulness of OCT-based classification of the PPA zones in distinguishing glaucomatous damage of the optic nerve from myopic damage in non-highly myopic eyes.

## Introduction

The association between glaucoma and the parapapillary atrophy (PPA) has long been recognized.[1] Studies have shown that the presence and the size of the PPA are significantly correlated with the presence,[2] severity,[3] and progression of glaucoma.[4–6] Although these reports suggest the relevance of the PPA in the diagnosis and monitoring of glaucoma, the PPA is influenced by several other factors such as age and myopia.[2,7–9] As these factors also increase the risk of glaucoma,[10–13] methodology to distinguish the influence of myopia and age on the PPA from that of glaucoma is necessary.

Traditionally, the PPA has been classified into a central alpha zone and a peripheral beta zone based on fundus photography.[1] The alpha zone is identified as an irregular hypo- and hyper- pigmentation of the retinal pigment epithelium (RPE), whereas the beta zone is characterized by atrophy of the RPE and the choroid. With the introduction of optical coherence tomography (OCT), a new classification of the classic beta zone PPA has been proposed.[14] The area without RPE but with Bruch’s membrane (BM) was defined as the beta zone, and the area with neither RPE nor BM was defined as the gamma zone. Studies have shown that the OCT-defined beta zone is associated with glaucoma, whereas the gamma zone is associated with myopia, but not with glaucoma.[14,15] Other studies have shown an association between OCT-defined beta zone and deterioration of the retinal nerve fiber layer (RNFL) thickness and visual field sensitivity.[16,17] These results suggest that OCT-based classification is more relevant than the conventional photograph-based classification in distinguishing the influence of glaucoma from that of myopia on the PPA.

Although these studies suggested the potential of the OCT-based new classification of the PPA zones in differentiating glaucomatous optic nerve damage from that of other causes, some other researches challenged these assumptions. For example, a recent study showed that neither the OCT-defined beta nor the gamma zone significantly correlated with glaucoma in eyes with myopia.[18] Furthermore, the association between the OCT-defined PPA zones and age remains controversial; one study showed that age positively associated with the gamma zone area,[14] whereas another study reported the association between age and the presence of the beta zone.[15]

Disagreements among studies could be in part attributed to the difference in study population, in particular the definition of myopia. Vianna et al. described that the new classification may not be useful in myopic eyes (defined as a refractive error greater than -2 diopters); however, according to previous reports, there was significant difference in the morphology of the optic disc and the PPA between highly myopic eyes and low to moderate myopic eyes, whereas there was no significant difference between hyperopic eyes and low to moderate myopic eyes.[2,8] These previous investigations suggest that highly myopic eyes should be analyzed separately from non-highly myopic eyes in morphological studies of the optic nerve head complex. Subsequent studies have shown that the optic disc and the parapapillary atrophy start to enlarge at about a value of −8.00 diopters of refractive error or an axial length of 26.5 mm.[19,20] Therefore, cut-off values of -8 diopters and 26.5mm have been proposed to define high myopia.[21]

Another possible cause for the conflicting results among studies may lie in the methodology for measuring the PPA. In previous studies, areas and widths of the PPA zones were measured on the near-infrared (IR) or color fundus images.[14,15,18] The borders of the PPA zones were first identified in the B-scans, and subsequently mapped onto the corresponding near-infrared (IR) or color fundus images using simultaneous visualization. Then the areas and the lengths of the zones were measured on the corresponding fundus images. However, mapping of the borders on corresponding fundus images are sometimes challenging, especially in eyes with optic disc distortion that is commonly observed in myopic eyes.[22,23]

We conducted the current study to clarify the association between the OCT-defined PPA zones and glaucoma, myopia, and age. We measured areas of the PPA zones in en face reconstructed images extracted from three dimensional (3D) swept-source OCT (SS-OCT) volumetric datasets. This method offers easy and reliable detection of the PPA zones, even in eyes with optic disc distortion. Considering the morphological distinction between highly myopic discs and non-highly myopic discs, we excluded eyes with high myopia, defined as ≤ −8.0 diopters of refractive error or ≤ 26.5 mm of an axial length. Multivariable regression analyses were used to investigate the associations between areas of the OCT-defined PPA zones and clinical factors such as glaucoma, axial length, and age, while adjusting for possible confounding effects of other factors. The approach we have used in the current study aims to establish the clinical significance of OCT-defined PPA zones in non-highly myopic eyes.

## Materials and methods

### Study participants

This study included consecutive subjects with glaucoma and healthy control subjects who underwent SS-OCT imaging at the Department of Ophthalmology, Osaka University Hospital. Glaucoma (G group) was diagnosed based on the presence of glaucomatous optic neuropathy (localized or diffuse neuroretinal rim thinning and/or retinal nerve fiber layer defect) and an associated visual field defect. Visual fields were considered abnormal if (1) a glaucoma hemifield test value was outside the normal limits; and (2) at least 3 vertical, horizontal or diagonal contiguous test points in the same hemifield on the pattern deviation probability plot at P<5%, with at least 1 point at P<1%, excluding points directly above or below the blind spot; or (3) a pattern standard deviation of less than 5% of the normal reference values. Age-matched control subjects (C group) were recruited from among the staff and employees of the hospital based on the following criteria; an IOP of 21 mmHg or lower, and normal appearance of the ONH on fundus examination. Exclusion criteria included high myopia defined as a refractive error of -8 diopter or greater or an axial length of 26.5 mm or longer, the presence of any other ocular or neurologic disorder that could cause visual field defects, a gonioscopically closed angle, secondary causes of IOP elevation, an anterior segment disorder or media opacity that affected image quality, and a history of intraocular surgery except uncomplicated cataract surgery.

All subjects underwent comprehensive ophthalmic examinations including measurement of the spherical equivalent refractive error using an auto refractometer (Nidek, Gamagori, Japan), measurement of the best-corrected visual acuity, Goldmann applanation tonometry, slit-lamp biomicroscopy, gonioscopy, dilated fundus examination, color photography of the optic disc (TRC-50DX, Topcon, Tokyo, Japan), and OCT. All subjects in the G group underwent SITA standard 30–2 visual field tests (Humphrey Field Analyzer; Carl Zeiss Meditec, Dublin, CA).

The protocol of this study was approved by the institutional review board of Osaka University Hospital. Each participant provided informed consent after explanation of the nature and possible consequences of the study. All study procedures adhered to the tenets of the Declaration of Helsinki for research involving human subjects.

### Swept-source OCT

A commercially available SS-OCT device (DRI-OCT, Topcon, Tokyo, Japan) was used to image the ONH and the parapapillary area. Axial and transverse resolution in tissue is 8 μm and 20 μm, respectively. The center wavelength is 1050 nm, and the scanning speed is 100,000 axial scans per second. With deeper penetration and higher scan speed, this machine is suitable for en face imaging, especially of deep structures. Eyes were imaged using the 3D scan mode, of which the A-scan density was 512 lines (horizontal) x 256 lines (vertical), within the scan time of 1.3 seconds. Poor quality images such as poor contrast images due to media opacity, or poorly fixated images were excluded.

### Analyses of parapapillary atrophy

**Fig 1:**
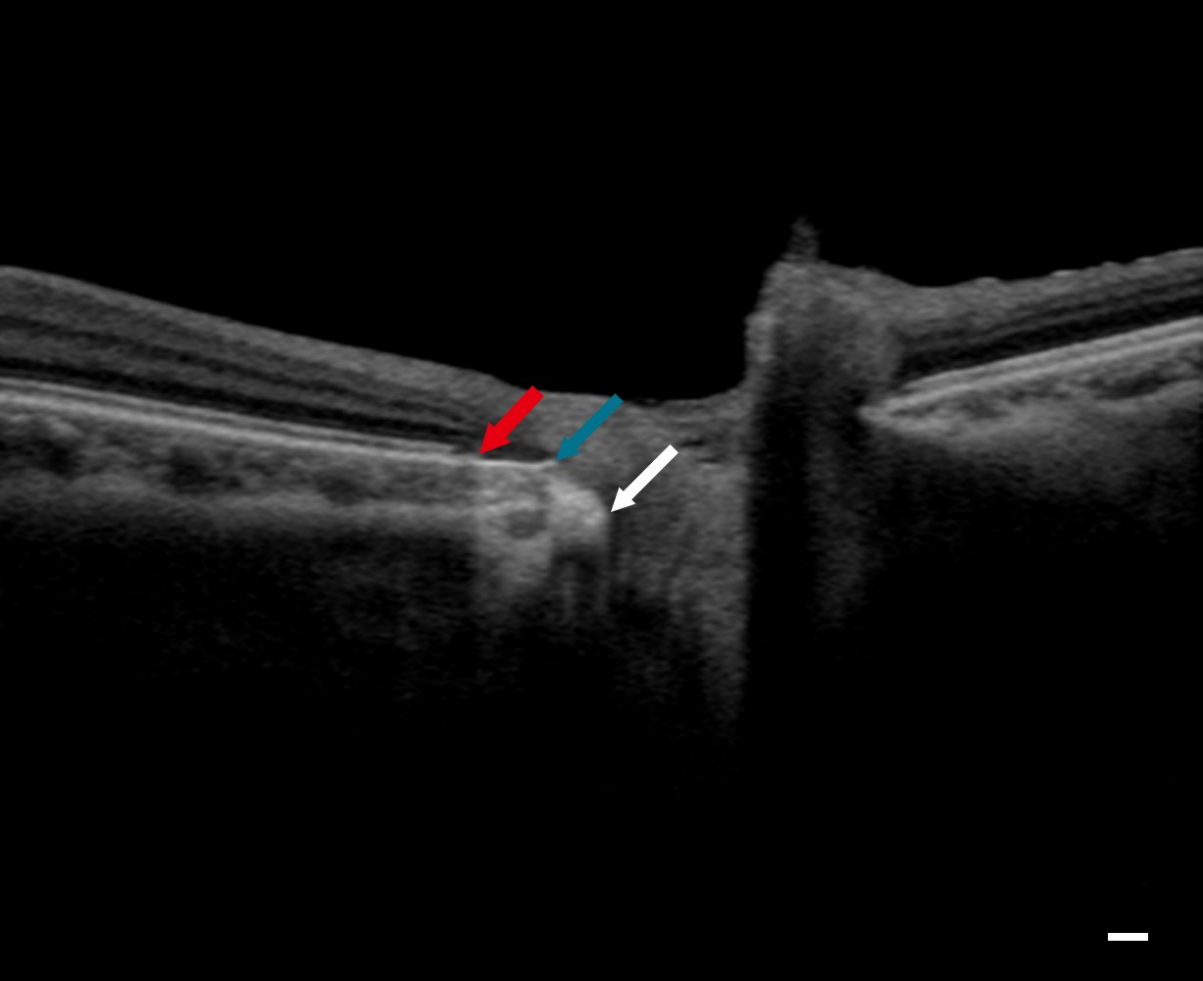
Identification of the beta zone and the gamma zone in a B-scan image. Scleral canal opening (white arrow), the edge of Bruch’s membrane (blue arrow), and the edge of the retinal pigment epithelium (red arrow) can be identified in a B-scan. A region between the termination of the retinal pigment epithelium and the termination of Bruch’s membrane is the beta zone, and a region between the termination of Bruch’s membrane and the scleral canal opening is the gamma zone. Scale bar = 200 μm.

In this study, the classic beta zone was divided into the beta zone and the gamma zone based on previous studies.[14,15] The beta zone is defined as an area without RPE, but with BM. The gamma zone is defined as an area with neither RPE nor BM. Representative images of the beta and the gamma zone are shown in the Fig 1.

**Fig 2:**
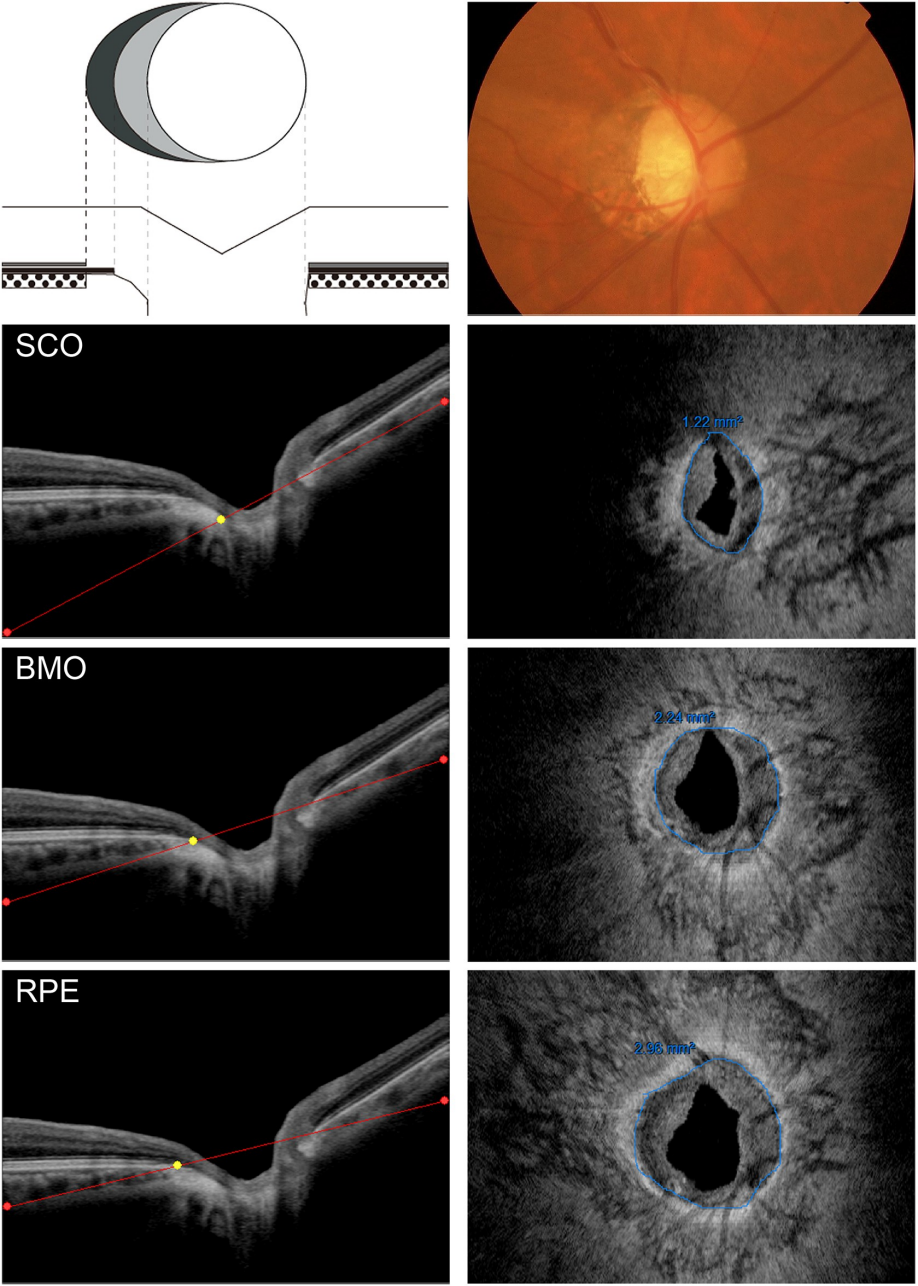
Measurements of the PPA zones. Top) Left panel illustrates the OCT-defined PPA zones. White circle in the above schema represents the optic disc. Grey area around optic disc (bare sclera without overlying Bruch’s membrane) is the gamma zone. Dark grey area (Bruch’s membrane without overlying RPE) represents the beta zone. It is difficult to distinguish between the beta zone and the gamma zone on the photograph (right panel). Second from top) Reference line was placed at the level of the SCO (red line) in a horizontal OCT scan of the same eye. Right panel shows a corresponding en face image. The SCO can be identified as a border between highly reflective sclera and mid-intensity neural tissue (blue line). Third from top) Reference line was placed at the level of the termination of Bruch’s membrane. BMO can be seen as a border between highly reflective Bruch’s membrane and mid-intensity neural tissue in an en face image (right panel). Bottom) Reference line was placed at the level of the termination of RPE. RPE tip can be identified as a border between high intensity RPE and low intensity neural tissue.

For calculating the areas of the beta zone and the gamma zone PPA, en face images were generated using image processing software EnView (Topcon, Fig 2 and S1 Fig). Anonymized image data were exported from an OCT instrument to another computer and assessed using EnView. En face slices at any desired depth, thickness, and angle can be obtained with the slanting application of the software. Using simultaneous visualization of B-scan images and en face images, a reviewer identified the landmark points such as the scleral canal opening, the end of BM, and the end of RPE in B-scan images. When measuring the areas of the scleral canal opening (SCO), we used this application to slant the reference line as to pass through both edges of the scleral canal in a horizontal B-scan image and a vertical reconstructed image. In an en face image obtained in this manner, SCO can be identified as a border between highly reflective sclera and moderately reflective neural tissues. The areas of SCO were measured by manually tracing the border of the SCO in en face images using the built-in caliper tool. The BMO area and the RPE-free area (the area bounded by the edge of RPE) were measured in en face images at the level of the BMO and the edge of the RPE, respectively. The beta zone PPA area was calculated as RPE free area minus BMO area, and the gamma zone PPA area was calculated as BMO area minus SCO area. Ocular magnification effect was adjusted based on the modified Littmann method with the refractive error, corneal radius, and axial length. All the images were reviewed and analyzed by a masked glaucoma specialist (AM). To validate the reproducibility of the measurements, 50 images were randomly selected and reexamined on a different day. The intraclass correlation coefficient was calculated for each measurement (SCO, BMO, and RPE free area) to evaluate the agreement between two measurements.

### Statistical analysis

Univariate and multivariate mixed-effects models were established for each PPA area as an outcome and with clinical characteristics such as gender, age, axial length, and glaucoma as predictors. Mixed effects modeling was used to deal with possible influence of including both eyes from one subject. We also analyzed the relation between the absence or presence of each PPA zone and clinical characteristics, as described in some previous reports.[15,16] Participants were divided into four categories: intact BM, discontinuous BM, lacking BM, and no PPA. Differences in continuous variables among groups were compared using a one-way analysis of variance, and categorical variables were compared using a Chi-square test. P value less than 0.05 was considered statistically significant in this study.

## Results

Originally, 98 eyes of 60 subjects were enrolled. Five eyes of 4 subjects were excluded from the study because of poor image quality. As a result, 93 eyes of 56 subjects were included in the analysis. Fifty eyes of 30 subjects were categorized in the G group, and 43 eyes of 29 subjects were categorized in the C group. The baseline characteristics of the subgroups are summarized in Table 1. Six eyes of 4 subjects were pseudophakic and others were phakic. No significant differences in age or gender were seen among the groups. Average ± standard deviation area of the beta and the gamma zone was 0.64 ± 0.79 and 0.16 ± 0.30 mm^2^, respectively. Average area of the beta zone in the G and C groups was 0.96 ± 0.89 and 0.27 ± 0.45 mm^2^, respectively. Mean area of the gamma zone in the G and C groups was 0.23 ± 0.33 and 0.07 ± 0.25 mm^2^, respectively.

**Table 1:** Baseline characteristics of the groups.

	Glaucoma (G)	Healthy control (C)	Total	P-value
Eyes / Subjects	50 / 30	43 / 26	93 / 56	
Age (years)	59.8 ± 9.5	60.1 ± 15.4	59.9 ± 12.4	0.938
Gender (F/M)	21/ 9	23 / 3	44 / 12	0.009
IOP (mmHg)	13.9 ± 2.2	15.5 ± 2.7	14.6 ± 2.5	0.009
Spherical equivalent (D)	-2.4 ± 2.3	-0.1 ± 0.1	-1.5 ± 2.2	0.003
Axial length (mm)	24.8 ± 0.9	23.5 ± 0.6	24.2 ± 1.0	<0.001

P-value calculated with mixed-effects modeling for numeric variables and the chi-square test for categorical variables.

**Fig 3:**
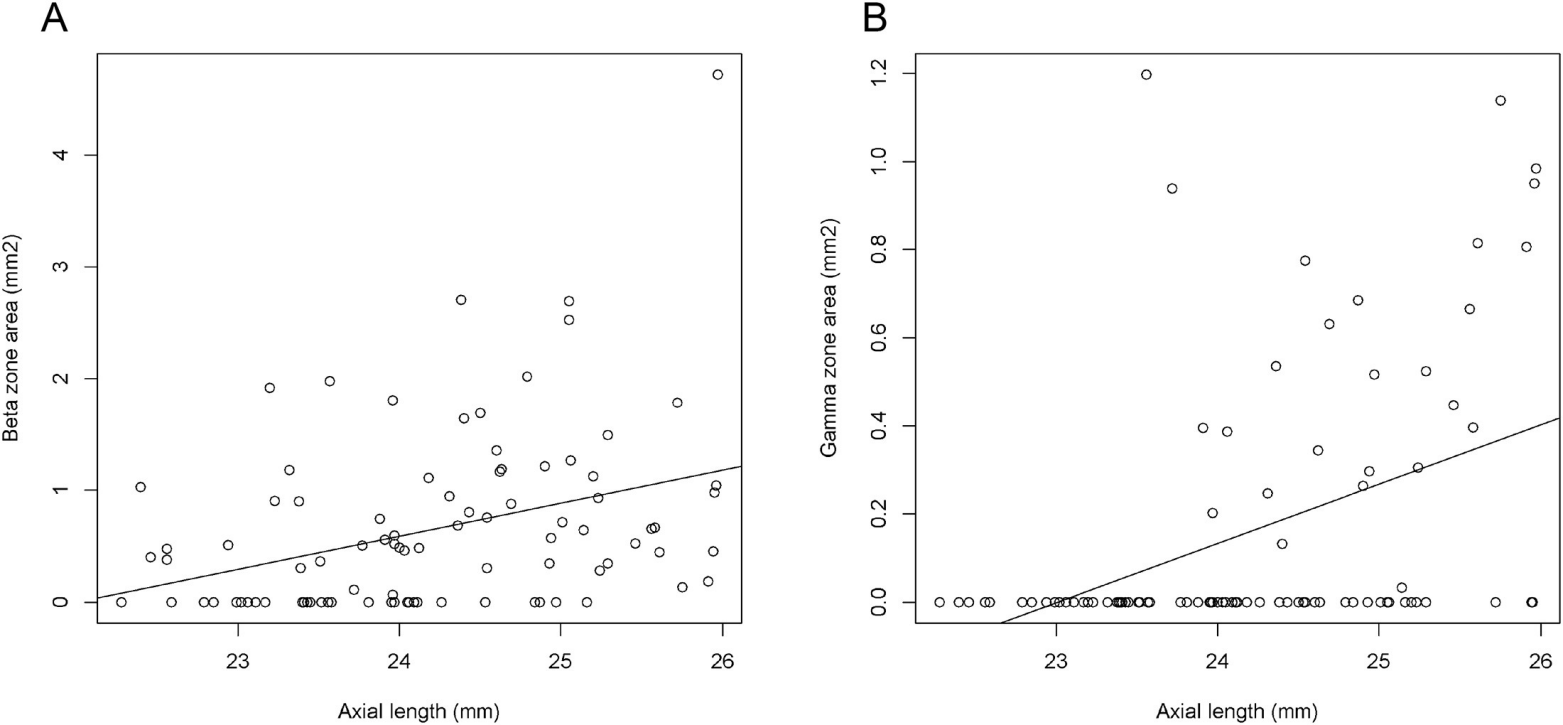
Scatterplots showing the correlation between axial length and A) the beta and B) the gamma zone parapapillary atrophy area.

Results of the univariate regression analyses evaluating the correlation between each PPA area and baseline characteristics are shown in Table 2. The beta zone PPA area significantly correlated with both glaucoma (P < 0.0001) and axial length (P = 0.0011, Fig 3). Age and gender did not significantly correlate with the beta zone area. The gamma zone PPA area significantly correlated with axial length (P = 0.0001). Gender, age, and glaucoma did not significantly correlate with the gamma zone area.

**Table 2:** Univariate regression analyses between patient characteristics and the beta zone and the gamma zone PPA area.

Beta zone area	Value	SE	t	P-value
Male Gender (vs Female)	0.4962	0.2219	2.236	0.0304
Age	0.0149	0.0074	1.998	0.0519
Axial Length	0.3027	0.0873	3.466	0.0011
Glaucoma (vs control)	0.6955	0.1553	4.478	< 0.0001
**Gamma zone area**				
Male Gender (vs Female)	-0.0027	0.0995	-0.027	0.9786
Age	-0.0047	0.0032	-1.459	0.1505
Axial Length	0.1435	0.0343	4.187	0.0001
Glaucoma (vs control)	0.1797	0.0783	2.294	0.0257

PPA: parapapillary atrophy

Results of the multivariate regression analyses evaluating the correlation between each PPA area and baseline characteristics are shown in Table 3. The beta zone area showed significant positive correlation with age (P = 0.0249) and glaucoma (P = 0.0139). Correlation between axial length and the beta zone area did not reach statistical significance in a multivariate model (P = 0.2106). Gamma zone area showed significant positive correlation only with axial length (P = 0.0011). In addition to the mixed effects analyses, Kendall’s univariable rank correlation analyses were also performed. Kendall’s Tau coefficient confirmed the positive correlation between axial length and the beta zone area (S1 Table).

**Table 3:** Multivariate regression analyses between patient characteristics and the beta zone and the gamma zone PPA area.

Beta zone area	Value	SE	t	P-value
Intercept	-3.8713	2.6313	-1.471	0.1482
Male Gender (vs Female)	0.1442	0.1993	0.723	0.4741
Age	0.0149	0.0063	2.337	0.0249
Axial Length	0.1367	0.1076	1.270	0.2106
Glaucoma (vs control)	0.5094	0.1967	2.590	0.0139
**Gamma zone area**				
Intercept	-3.3087	1.0941	-3.024	0.0034
Male Gender (vs Female)	-0.1093	0.0945	-1.156	0.2529
Age	-0.0275	0.0030	-0.909	0.3676
Axial Length	0.1513	0.0447	3.385	0.0011
Glaucoma (vs control)	0.0064	0.0909	0.071	0.9437

PPA: parapapillary atrophy

Results of the categorical analyses are shown in Table 4. There was no significant difference in gender or age among categories. Axial length was longer in groups that contained the gamma PPA (discontinuous BM group and lacking BM group). Glaucoma was more prevalent in groups that contained the beta PPA (intact BM group and discontinuous BM group).

**Table 4:** Clinical characteristics by subgroup categorized based on presence or absence of beta zone and gamma zone PPA.

	Intact BM	Discontinuous BM	Lacking BM	No PPA	P-value
Gender (F/M)	29 / 10	17 / 5	3 / 1	23 / 5	0.0901^1^
Age	64.3 ± 10.0	56.1 ± 10.3	50.1 ± 14.0	59.5 ± 15.0	0.0210^2^
AL	24.2 ± 1.0	25.0 ± 0.7	24.4 ± 0.7	23.5 ± 0.7	<0.0001^2^
Glaucoma (yes/no)	25 / 14	20 / 2	2 /2	3 / 25	<0.0001^1^

^1^: chi-square test,

^2^: one-way anova

P values show the significance of comparisons among categories.

BM: Bruch’s membrane, PPA: parapapillary atrophy, AL: axial length

The ICC for the measurements of the SCO area, the BMO area, and the RPE area were 0.86, 0.79, and 0.87, respectively. These values can be interpreted as fair to good reproducibility.[24]

## Discussion

OCT-based subclassification of classic beta zone PPA into the beta zone and the gamma zone has shown promise in differentiating glaucomatous optic nerve damage from myopic optic nerve damage.[14,15] Recent research questioned the relevance of OCT-based PPA classification in myopic eyes, defined by the authors’ own cut-off value (-2 diopters).[18] However, the cut-off value of -8 diopters of refractive error or 26.5 mm of an axial length has been proposed for defining high myopia based on hospital-based and population based studies.[19,20] Therefore, we conducted the current research in a population without high myopia, defined by this evidence-based criteria. In addition, we measured PPA areas in en face images to facilitate clear detection of the PPA zones, even in eyes with optic disc distortion, which are commonly seen in myopic eyes. Results of the current study showed that the beta zone area positively correlated with glaucoma and age, whereas the gamma zone positively correlated only with axial length in multivariable models. These findings extend those of previous studies, confirming that the beta zone is correlated predominantly with glaucomatous optic nerve damage, while the gamma zone is correlated more strongly with myopic optic nerve deformation[14,15,25] This study confirmed the relevance of the new PPA classification in distinguishing glaucomatous optic nerve damage from myopic optic nerve damage, at least in non-highly myopic subjects. In myopic eyes, diagnosis of glaucomatous optic neuropathy with fundus photographs is often difficult due to morphological changes of the optic nerve head associated with myopia such as large disc[8,26] and disc tilt.[22,27] Evaluation of retinal nerve fiber layer damage by OCT is also challenging due to measurement error in myopic eyes because of morphological changes of the optic nerve head.[23,28] OCT-defined subclassification of the PPA zones holds promise as a new tool for diagnosing glaucoma in myopic eyes. Further study is warranted to evaluate the relevance of this method in eyes with high myopia.

Age positively correlated with the area of the beta zone in this study. This is in line with the results of the study by Kim et al,[15] but conflicts with those of Dai et al and Vinna et al.[14,18] It is quite challenging to evaluate the impact of age on the PPA zones separately from those of myopia and glaucoma, because of very strong correlation of age with both glaucoma and myopia. It is well known that older age is associated with increased risk of glaucoma.[11,13] Also, significant increase of myopic refractive errors in young population has been reported globally.[29,30] Therefore, further study is necessary to clarify the complex relationship between age and the PPA zones while adjusting for possible confounding effects of glaucoma and myopia. In addition, the protective effect of the gamma zone against glaucoma in a previous study[14] was not confirmed in this study and requires further investigation.

Some previous investigators performed categorical analyses assessing the relationship between clinical characteristics and presence or absence of each PPA zone.[15–17] Therefore, we also performed categorical analyses. Prevalence of glaucoma was largest in the discontinuous BM group, followed by the intact BM group, lacking BM group, then the no PPA group. Axial length was longer in intact BM and discontinuous BM groups than in the lacking BM and the no PPA group in this study, which is in agreement with the previous report.[15] Age was the oldest in the intact BM group, followed by discontinuous BM group, no PPA group, then the lacking BM group. This is similar to the result of the previous study.[15] Together, results of the categorical analysis in this study is generally in agreement with the previous investigation.

In this study, we measured the areas of PPA zones using en face images reconstructed from 3D volume. In previous OCT studies, area[14] and width on horizontal scan[15,17] of the PPA zones were measured and used for correlational studies. We measured area instead of width of the PPA because we assume the area reflects the overall severity of the PPA zones better than the width on single to several scans in horizontal quadrant. In previous investigations, areas and widths of the PPA zones were measured on the IR image or the color fundus image, which can be considered as projection images of target structures projected onto a plane that is perpendicular to the OCT scan beam. Areas and widths measured on these images, therefore, differ from those of the real structure depending on the angle between the scan beam and the target structure. We measured the dimensions of target structures on en face slices generated as to pass through the surfaces of target structures. This method is relatively unsusceptible to measurement errors due to disc tilt. The ICC for the measurements of the SCO area, the BMO area, and the RPE area ranged from 0.79 to 0.87, which indicates high precision of this measurement method.[24] If necessary, widths may also be measured reliably with this method. We used an SS-OCT system with a center wavelength of 1050nm and the scanning speed of 100,000Hz to generate en face images. A longer wavelength of the light source enables higher penetration into deeper tissues which provides clear view of the parapapillary structures.[31–33] A faster scan speed of the SS-OCT machine facilitates acquisition of dense sampling over a broad area that is necessary for three-dimensional imaging.[31] With these features, the SS-OCT device is well suited for generating en face images of the parapapillary structures.

There are several limitations to the current study. We could not evaluate the association between PPA zones and glaucoma progression[16,17] because of the cross-sectional design of the study. Also, it is not clear whether the results of this study apply to populations other than Japanese. Nevertheless, this is the first study to measure the OCT-defined beta and gamma PPA zones using the en face images.

In conclusion, en face images of the parapapillary structures were successfully generated from three-dimensional SS-OCT data which facilitate measurements of the beta and the gamma PPA zones. Glaucoma is significantly associated with the beta zone but not with the gamma zone. Myopia is significantly associated with the gamma zone. Some findings in previous studies such as the protective effect of the gamma zone against glaucoma or the association between age and the gamma zones were not confirmed in this study and warrant further investigation. Results of the current study clarified the clinical usefulness of OCT-based classification of the PPA zones in distinguishing glaucomatous damage from myopic damage, at least in non-highly myopic eyes, including eyes with low to moderate myopia.

## Supporting information

S1 Fig Measurements of the PPA areas on en face OCT images. (TIF)

S1 Table Results of the Kendall rank correlation analysis. (DOCX)

S2 Table Baseline demographics of the whole participants. (CSV)
