# Supplementary figures and images for: Measurements of the parapapillary atrophy zones in en face optical coherence tomography images
Source: PLoS One. 2017 Apr 17;12(4):e0175347. doi: 10.1371/journal.pone.0175347 (PMC5393576; doi:10.1371/journal.pone.0175347)

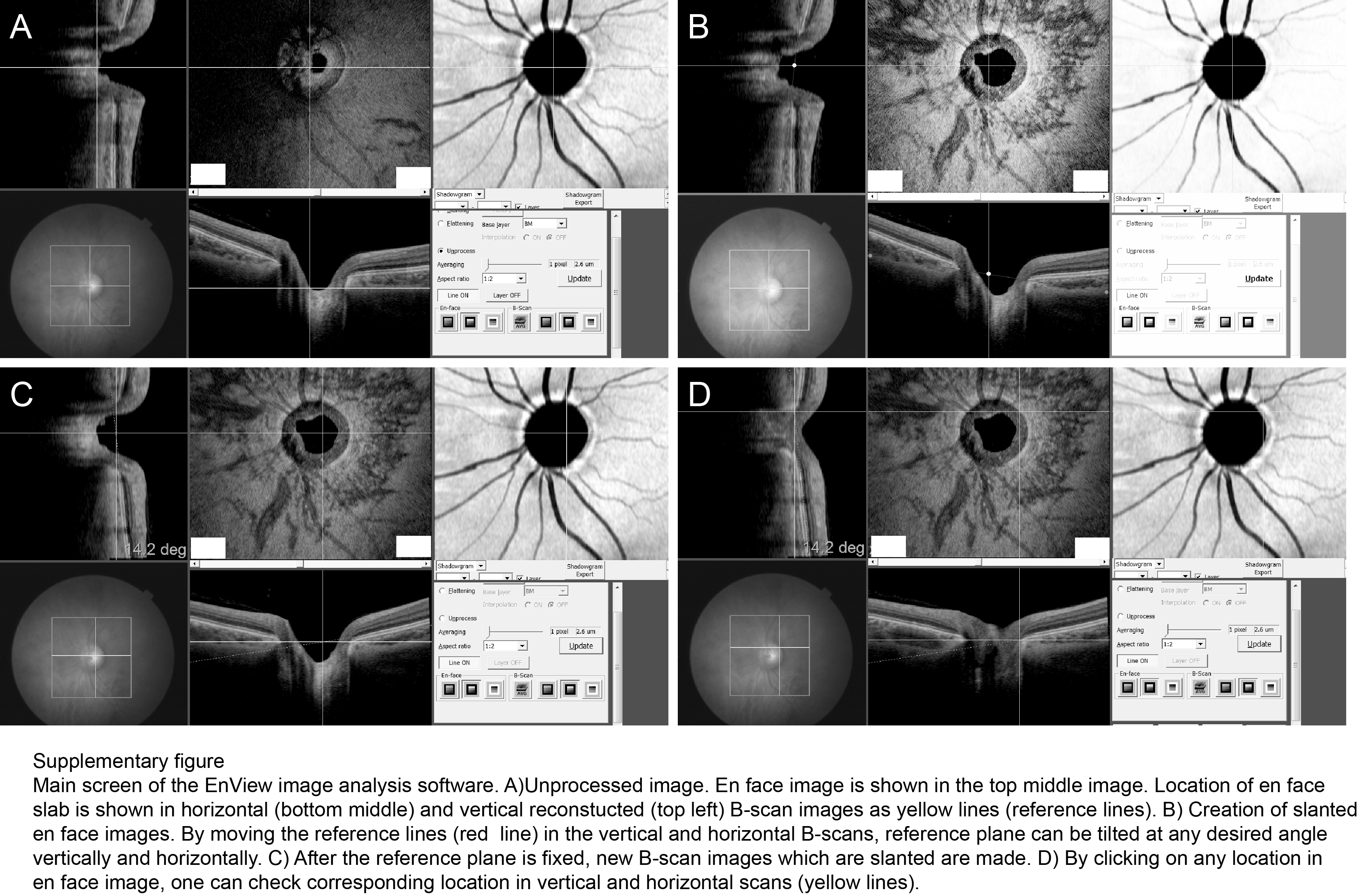

Supplement: S1 Fig — (TIF) [file pone.0175347.s001.tif]
