# Supplementary material for: Measurements of the parapapillary atrophy zones in en face optical coherence tomography images
Source: PLoS One. 2017 Apr 17;12(4):e0175347. doi: 10.1371/journal.pone.0175347 (PMC5393576; doi:10.1371/journal.pone.0175347)
Supplement: S1 Table — (DOCX) [file pone.0175347.s002.docx]

# Results of the Kendall rank correlation analysis

|  | **Tau** | **z** | **P-value** |
| --- | --- | --- | --- |
| **Beta zone area** |  |  |  |
| Age | 0.118104 | 1.6041 | 0.1087 |
| Axial Length | 0.276811 | 3.7706 | 0.00016 |
| **Gamma zone area** |  |  |  |
| Age | -0.23203 | -2.8871 | 0.003888 |
| Axial Length | 0.373422 | 4.66 | 0.000003 |
